# Predictors of Treatment Adherence in a Randomized Clinical Trial of Digital Therapeutics with Pharmacology for Alcohol Use Disorder (AUD)

**Published:** 2025-08-08

**Authors:** Morris D. Bell, Yarani Gonzalez, Brian Pittman, Gihyun Yoon

**Affiliations:** 1 Department of Psychiatry, Yale School of Medicine, New Haven, CT, USA; 2 VA Connecticut Healthcare System, West Haven, CT, USA

**Keywords:** Alcohol use disorder, Treatment adherence, Digital therapeutics, Predictors, Randomized clinical trial

## Abstract

**Background/Aims:**

Non-adherence is a significant issue in clinical trials of new therapeutics; and Alcohol Use Disorder (AUD) studies are particularly vulnerable to discontinuation. Moreover, clinical trials involving behavioral intervention over time may be even more difficult to complete. Digital therapeutics make participation in treatment much more accessible and may therefore reduce one of the barriers to study participation. This study aims to investigate predictors of treatment adherence in a clinical trial that collected data over 13 weeks involving a digital therapeutic Cognitive Remediation Therapy (CRT) and donepezil for patients with AUD, focusing on (1) CRT adherence (total hours completed); (2) medication adherence (pill count) and (3) total adherence that combines 1 and 2.

**Methods:**

Baseline data including demographics, illness characteristics, cognitive assessments, self-reports of functioning and disability, psychiatric symptoms and personality traits were collected on 52 participants. Exploratory analyses included parametric and non-parametric analyses of baseline variables to predict adherence over 13 weeks.

**Results:**

Of the 52 participants who were randomized, 49 completed baseline assessments and entered the study. The mean CRT hours was 29.49 hours (SD=27.90) out of a possible 65 hours (45.37%). Mean weekly medication adherence was 48.78 (SD=38.81) out of 84 pills (58.02%). Medication adherence was correlated with younger age and Total Adherence was negatively correlated with WHODAS 2.0 Self-care indicating that greater adherence is associated with lower self-care dysfunction. Medication adherence was significantly related to physical ability on the VR-12 but with none of the other variables. CRT adherence was not directly related to any baseline variables. A linear regression analysis was performed to predict Total Adherence. A significant model was obtained with WHODAS 2.0 Self-care and Age as significant variables. No relationship was found between Self-care and substance abuse or craving variables, or with cognitive measures, but there were some significant relationships with psychiatric symptoms and relatedness variables. A linear regression of Self-care Predictors produced a significant model with Alienation as the only predictor.

**Conclusions:**

This study showed a relatively high rate of adherence: over two-thirds of participants completed the 13-week protocol, with substantial engagement in both digital therapeutics and medication. The findings highlight the importance of Self-care, as a predictor of adherence, whereas illness-specific characteristics did not predict adherence, suggesting that adherence may not vary by AUD characteristics. While Self-care was the best predictor of Total Adherence, we found that psychiatric symptoms and relatedness variables were predictors of Self-care. Regression analysis indicates that Alienation captures the most variance in Self-care, suggesting the importance of therapeutic alliance in clinical trials.

## INTRODUCTION

Alcohol Use Disorder (AUD) is a chronic, relapsing addictive disorder [1]. Therefore, successful treatment outcomes require consistent adherence to AUD treatment [2]. However, treatment adherence remains a significant challenge in clinical practice with up to 50% of patients across all medical fields failing to adhere to prescribed medications and non-adherence rates are even higher among those with AUD [3,4]. Non-adherence is also a significant issue in Randomized Clinical Trials (RCTs) of new therapeutics which depend upon adequate adherence rates; and AUD studies with outpatients are particularly vulnerable to discontinuation, making generalization of findings more difficult. A review of phase 2 and 3 clinical trials for AUD registered on ClinicalTrials.gov found that 39.1% were discontinued or never published with failed adherence a common reason for discontinuation, despite contingency management and financial incentives [5].

While some clinical trials with a brief intervention and follow-up may be intrinsically prone to less non-adherence, those requiring longer interventions and follow-up times may experience less adherence due to trials demands (e.g., repeated in-person behavioral session), scheduling conflicts and other life demands. A significant advantage of digital therapeutics is the convenience and accessibility they offer to participants. However, a review of 4 FDA-authorized neuropsychiatric digital therapeutic clinical trials found generally high attrition and low engagement [6]. Included among these was a trial examining the effects of combined contingency management and internet behavioral skills training in promoting prosocial reinforces for substance use disorder treatment [7]. While the study had 76.3% engagement, all sessions were supervised by a live therapist in the clinic, so it may not be relevant to the question of whether remote digital therapeutics may improve adherence.

Internet access and the use of remote devices have increased dramatically since 2014. Therefore, treatment access among those with psychosocial instability and limited resources via the internet may be especially important for clinical trials among AUD. Such digital intervention could mitigate sampling bias by attracting a more diverse sample, thus increasing generalizability of findings.

A recent systematic review of adherence among 96 RCTs utilizing digital therapeutics for depression found that 44.2% of participants completed all the modules and that greater adherence to treatment was associated with better outcomes [8]. While studies with less than 10 days of digital therapy were excluded, the results did not account for variations in length of treatment. No other information was provided regarding predictors of adherence.

Several factors have been identified as predictors of treatment adherence in AUD in clinical practice. Poor adherence has been associated with sociodemographic variables such as younger age, lower education, unemployment and lack of independent housing [10–12]. Certain AUD-related characteristics, such as early exposure to alcohol and shorter abstinence duration at hospital admission, have also been linked to lower adherence [10].

The relationship between AUD severity and treatment adherence is complex. Severe AUD may hinder adherence due to cognitive impairments or withdrawal but can also increase adherence if patients are more motivated. Some studies report no clear link between adherence and AUD severity or baseline alcohol consumption, while others report that individuals who self-report high consumption at baseline are more likely to adhere to intervention over time [13–15].

Personality traits also play a role in treatment adherence. Traits such as impulsivity and sensation-seeking are associated with lower adherence, whereas conscientiousness is linked to better adherence [16–18]. Clear goal setting and specific intention to reduce drinking prior to intervention has been associated with improved engagement over time [15]. Cognitive impairments (e.g., deficits in memory, executive function and reward processing) can negatively impact treatment adherence in AUD [19,20]. These impairments are linked to deterioration in self-care and household functioning, both of which are increasingly recognized as important factors in treatment adherence in AUD treatment [21–26]. Self-care practices (e.g., physical exercise, mental wellness and self-efficacy) can enhance engagement and support sustained recovery [27].

These investigations into factors that affect treatment adherence for AUD outside of clinical trials suggest that they may be relevant to our examination of predictors of adherence in our RCT. However, we are unaware of any prior reports of predictors of adherence in AUD clinical trials involving digital therapeutics and thus our aims are exploratory and without specific hypotheses.

### Study aims:

This study is an exploratory examination of predictors of treatment adherence in a clinical trial involving a digital therapeutic and medications for patients with AUD, focusing on adherence to treatment in general and sub-analysis of two endpoints: (1) Cognitive Remediation Therapy (CRT) adherence (the digital therapeutic), measured by total hours completed, (2) medication adherence, measured by weekly self-reports and (3) total adherence that combines 1 and 2. The analysis is based on data from 52 participants in an ongoing clinical trial. Without unbinding the study, we aim to identify patient characteristics that predict adherence. Based on the literature, we explore demographic characteristics, AUD characteristics, personality traits, cognitive functioning and levels of self-reported disability as possible predictors.

## MATERIALS AND METHODS

### Participants

We conducted a retrospective analysis of data from 52 participants who completed an ongoing clinical trial, a National Institute on Alcohol Abuse and Alcoholism (NIAAA)-funded study (RO1AA029075) of cognitive training and donepezil in AUD [28]. This 13-week, randomized, double-blind, placebo-controlled study included four arms: donepezil+Cognitive Remediation Therapy (CRT), donepezil+sham CRT, CRT+placebo and sham CRT+placebo. Participants were male and female patients aged 18–80 years, diagnosed with current AUD via the Mini International Neuropsychiatric Interview (MINI) and reporting at least one heavy drinking day in the past 30 days [29]. Exclusion criteria included psychotic disorders and medical conditions likely to impair cognition (e.g., Parkinson’s, Alzheimer’s, Huntington’s chorea, or moderate/severe TBI).

The sample (N=52) was 63.5% male, with a mean age of 46.38 year (SD=14.90). 9.6% were U.S. Veterans. Mean years of education was14.69 (SD=3.13) with 71.2% with a college degree or higher. The sample was 57.7% White, 34.6% Black/African American, 7.7%. Other and 7% were Hispanic (White or Other). Average lifetime alcohol use to intoxication was 244.37 months (SD=180.53). All met DSM-V criteria for Alcohol Use Disorder and 42% had a secondary psychiatric diagnosis: 13.5% Major Depressive Disorder; 11.5% Marijuana Use Disorder, 9.6% Cocaine, Opioid or Other Use Disorder, 5.8% PTSD, 2% Antisocial Personality Disorder. Their Wechsler Test of Adult Reading (WTAR) mean score, used as an estimate of the pre-morbid IQ, was 109.88 (12.78) [30]. The above average pre-morbid IQ and the higher level of education was unexpected but may be related to digital recruitment strategies and the requirement that the individual have some experience using a computer or laptop.

### Procedures

Participants were recruited from the community with printed flyers and postings on social media developed by a service called Build Clinical and from clinician referrals. People who responded to the flyers or social media were screened by phone for eligibility and if qualified, they were invited for a written informed consent and HIPAA authorization meeting and baseline assessment for which they were provided compensation. These included diagnostic interview (MINI adapted to DSM-5), WTAR, physical exam and comprehensive psychosocial and demographic interviews to confirm eligibility; if eligible, they were also administered neurocognitive assessments and self-report instruments sometimes at a second visit. All interviews and testing were performed in private offices to ensure confidentiality. Participants were administered the clinical measures in accordance with approved protocols by the IRB at the VA Connecticut Healthcare System (VACHS) and Yale University and included US Veteran and non-Veteran samples.

Eligible participants were randomized equally into one of 4 conditions: Donepezil+CRT, (Brain HQ by Posit Science); donepezil+sham CRT (computer games hosted on the same Posit Science website); CRT+Placebo; Sham CRT+placebo. The main hypothesis was that CRT+Donepezil would be superior to the other conditions. A complete description of the protocol has been published elsewhere [28].

During the 13-week intervention, participants received weekly Timeline Follow-Back (TLFB) assessments [31]. Critical events and participation data were recorded. Cognitive testing occurred at baseline, week 7 and week 13. Medication (donepezil or placebo) was administered at 5 mg/day for four weeks, then 10 mg/day through week 13. All study staff, except the pharmacist, remained blinded to treatment condition. The current analysis was conducted without breaking the study blind, ensuring the integrity of the ongoing clinical trial.

### Measures

This study assessed predictors of (1) CRT or sham CRT adherence (total hours completed) and (2) weekly self-report of medication adherence (donepezil or placebo) corroborated by prescription refill at 28 days. In order to create an Adherence Total Score where medication adherence and CRT hours are weighed equally, the raw counts of hours of training and number of pills was summed for each subject and then categorized on a scale of 0 to 3 (Training Hours: 0=0, 1–25=1, 26–60=2 and 65=3; Pill Count 0=0, 1–17=1, 18–83=2, 84=3). The values for the categories was determined by balancing the number of participants in each category distribution while maintaining the anchor points of None (0) and Complete (60 training hours; 84 pills). The medication adherence and CRT adherence categories were then combined for a Total Adherence score from 0 to 6 (None=0, Poor=1, Partial=2, Good=3, Very Good=4, Excellent=5, Complete=6 Adherence). Predictor categories include: (1) demographics (e.g., age, gender); (2) AUD characteristics (e.g., AUD severity, baseline alcohol consumption, craving); (3) cognitive variables (e.g., attention, memory, executive function); (4) personality traits; and (5) self-care and household function.

Alcohol craving was measured with the Penn Alcohol Craving Scale [32]. Personality traits were assessed with the Bell Object Relations & Reality Testing Inventory [33]. Memory was measured via the Hopkins Verbal Learning Task (HVLT) and HVLT Delayed Recall, Digit Span [34,35]. BVMT, BVMT Delayed, Logical Memory I & II [36,37]. Attention was measured using the Integrated Visual and Auditory (IVA) Continuous Performance Test-2 (IVA-2) [38]. Executive function was measured using Wisconsin Card Sorting Test and Mazes Total Score [39,40]. Physical/mental health status was evaluated using the Veterans RAND 12 Health Survey (VR-12) [41]. Level of disability was measured by the WHO Disability Assessment Schedule (WHODAS) 36-Item Self-Report [42]. In the WHODAS 36-Item Self-Report, higher scores indicate greater disability or worse functioning in that domain. The BASIS-24 was used to measure psychiatric symptoms (e.g., depression, functioning, interpersonal relationships, self-harm, emotional lability, psychosis and substance abuse and overall score) [43].

### Participant compensation

Participants were paid $75 for baseline assessment procedures, $25 for 7-week testing follow-up and $75 for the 13-week comprehensive follow-up assessment. They were compensated $15/week for Time-Line Follow Back, $10 for weekly pill counts, $10 for their computer training orientation session and $5/hour for computerized training up to 65 hours. Total compensation for all procedures was $835, an amount deemed appropriate and comparable to other such studies as determined by the IRB of record.

### Statistical analysis

Given the exploratory nature of this study, we did not have a priori specific hypotheses. Descriptive statistics and correlation analyses (Pearson or Spearman, as appropriate) were used to characterize the sample and to examine potential associations between baseline characteristics and continuous measures of adherence: CRT hours, medication pill count and Total Adherence, Chi-Square and logistic regression were used to examine predictors of completion status (completed, did not complete). All tests were two-sided using an alpha=.05 significance threshold without correction for multiple comparisons in this exploratory study.

## RESULTS

Descriptive statistics for all variables used in the analyses below are presented in Supplementary Table 1.

### CRT and Medication adherence

**Figure 1:**
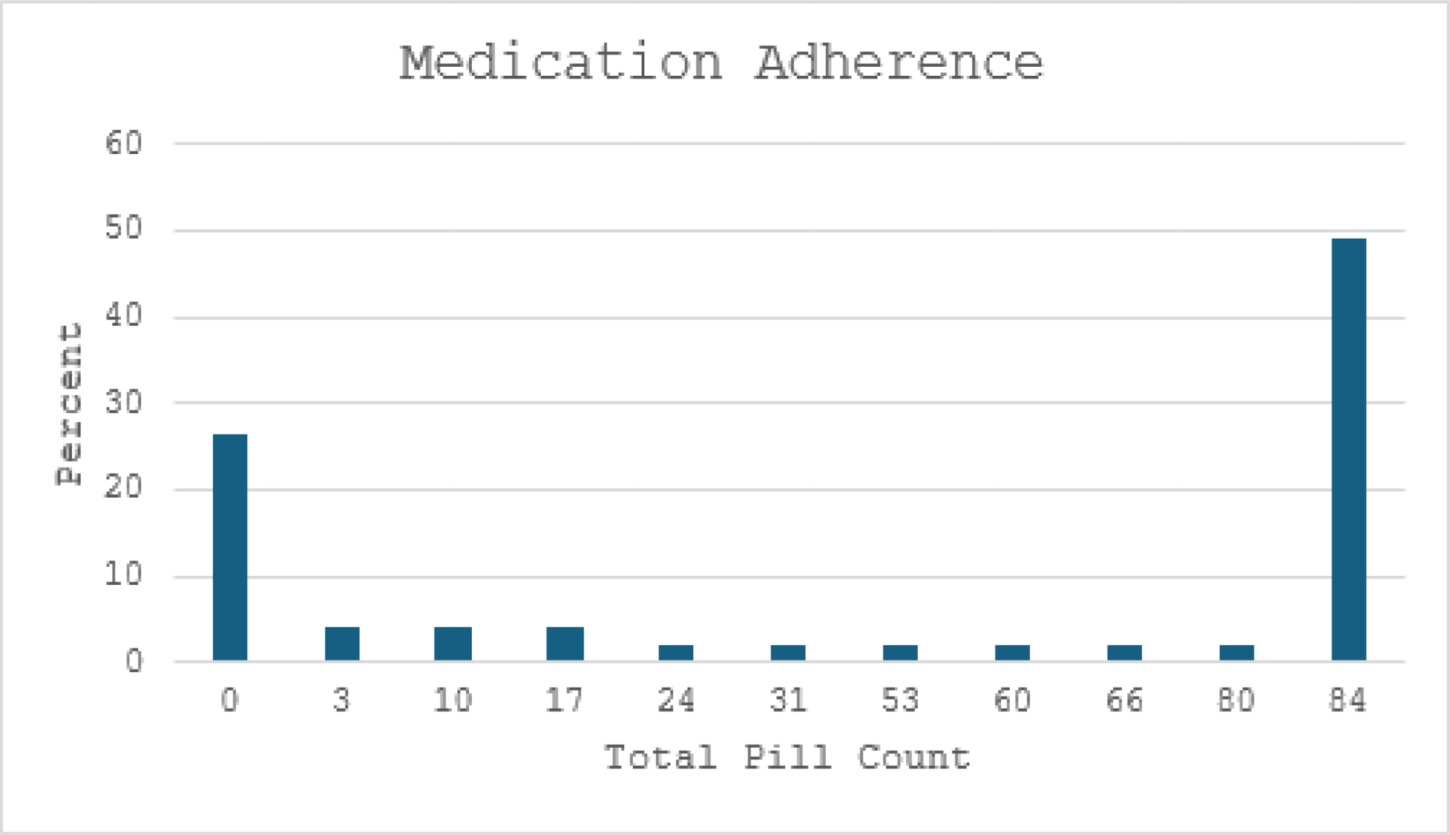
Percentage distribution of medication adherence.

**Figure 2:**
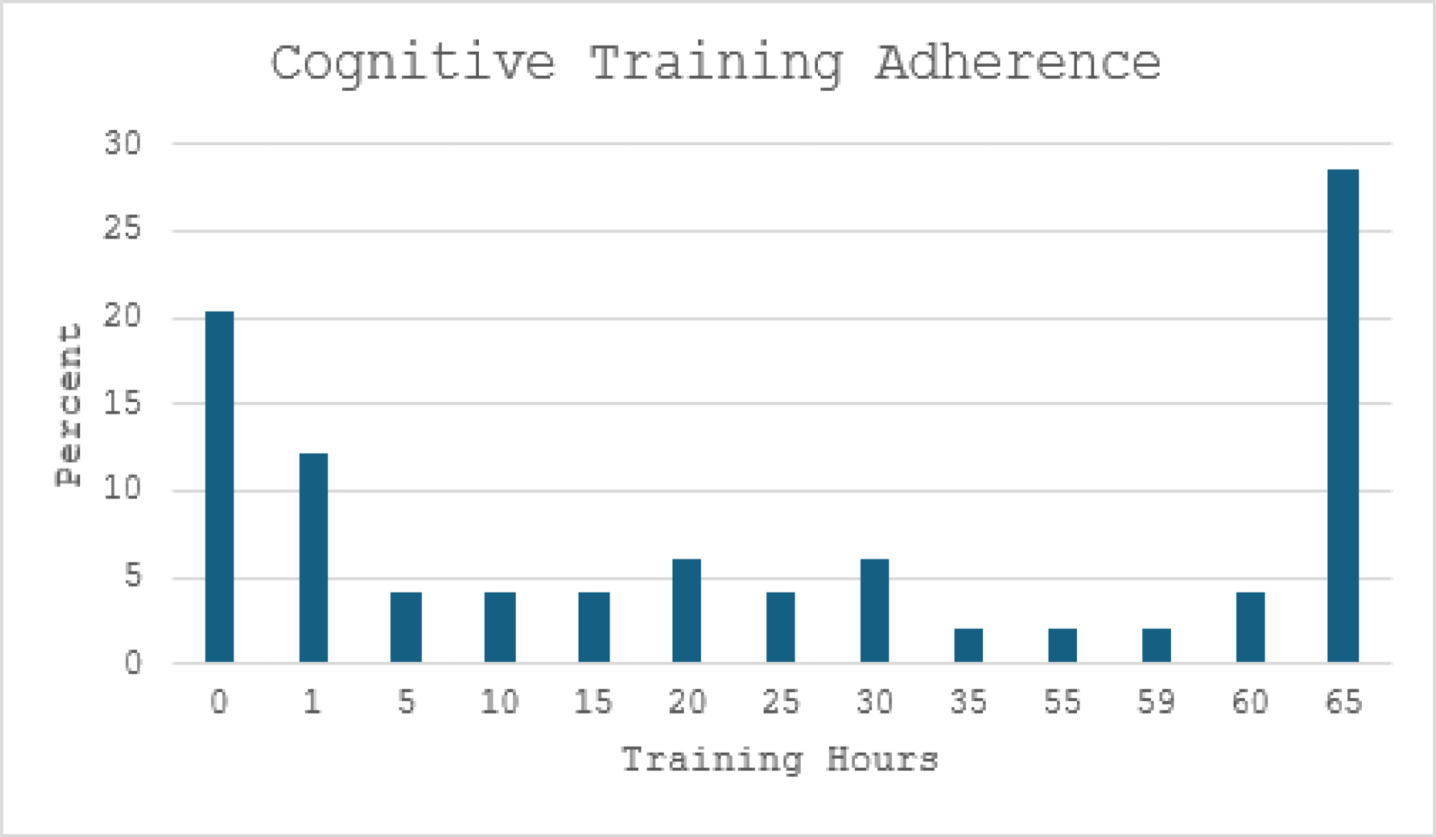
Percentage distribution of cognitive training adherence.

Of 52 participants who signed consent, 34 (65.4%) completed the study. Three participants were not randomized because they either withdrew before baseline assessment or did not meet inclusion/exclusion criteria. Removing the non-randomized individuals, there were 49 who entered the study and the completion rate was 69.4%. Among 49 participants, the mean CRT hours was 29.49 hours (SD=27.90) out of a possible 65 hours (45.37%); 56% completed 20+ hours, which may be regarded as a minimally adequate dosing. Mean weekly medication adherence was 48.78 (SD=38.81) out of 84 pills (58.02%). Distributions are shown in Figures 1 and 2. A higher percentage of participants adhered completely to medications (49%) than to CRT (28%) (Chi-square=4.30, df=1, p <.05), although cognitive training hours and medication adherence were significantly correlated (r=.561, p <.001).

**Figure 3:**
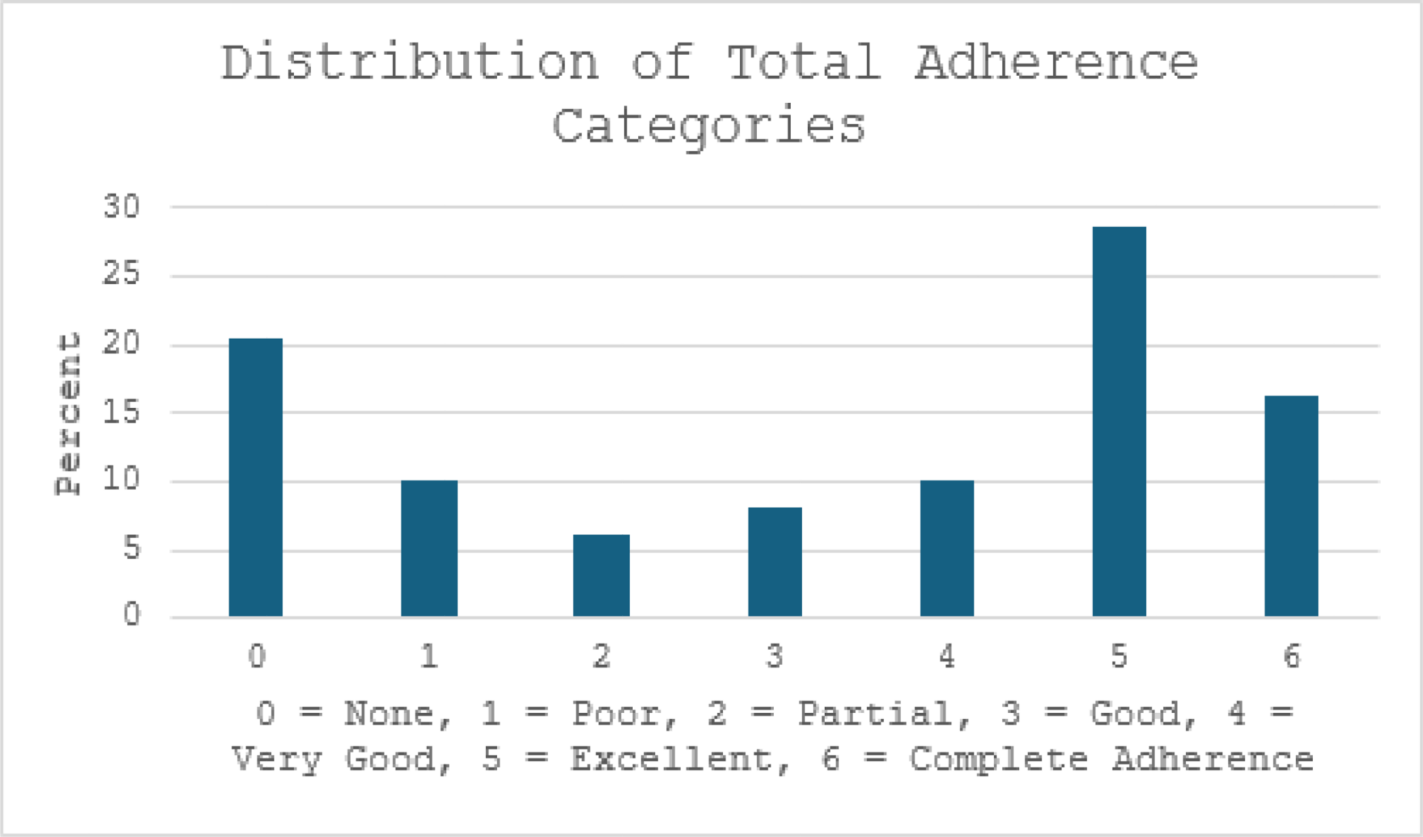
Percentage distribution of total adherence categories.

As described above, adherence categories were created for medication and CRT adherence and they were added together to provide a Total Adherence Score (mean=3.286, SD=2.245) which showed in Figure 3 that 55% had Very good, Excellent or Complete adherence. While CRT Adherence and Medication Adherence scores were not normally distributed and required non-parametric analysis (Spearman), Total Adherence met criteria for parametric analysis.

### Predictors of Adherence

Age, Education, Pre-morbid intelligence and Gender: No significant relationships emerged between Years of Education and CRT hours (r=.13, p=ns), medication adherence (r=.099, p=ns), or Total Adherence (r=.086, p=ns). Younger age was associated with better medication adherence (r=−.285, p <.05) but age was not correlated with Total Adherence (r=−.262, p=.069) or with CRT hours (r=−.172, p=.238). WTAR scores were also not associated with CRT hours (r=.042, p=ns), medication adherence (r=.069, p=ns), or Total Adherence (r=.070, p=ns). A Logistic Regression using Age, Education and WTAR to predict High Total Adherence (>4) or Low Total Adherence (<4) was not significant, correctly classifying only 61.4% of participants (Chi-sq=3.94, df=3, p=.268). No gender differences were observed with exactly 50% of men and women in the high and low Total Adherence groupings (Chi-sq.=0.00, df= 1, p=ns).

Alcohol craving, Lifetime alcohol use to intoxication and BASIS Alcohol and Drug symptoms (Table 2): Lifetime alcohol use to intoxication was significantly negatively correlated with Medication Adherence (−.338, p=.018) so that a longer history of intoxication was associated with poorer medication adherence; but since lifetime alcohol use is correlated with age (r=.520, p <.001), the correlation with Lifetime Alcohol Use to Intoxication was no longer significant when controlling for age (r=−.067, p=ns). No other correlations were significant between these predictors and any of the adherence measures.

No significant relationships (Supplementary Tables 2 and 3) were found between adherence measures and self-reported symptoms on BASIS-24 symptom subscales (BASIS Alcohol and Drug Abuse symptoms were reported above) or with Bell Object Relations and Reality Testing Inventory.

WHODAS 2.0 and VR-12 (Supplementary Table 4): Total Adherence was negatively correlated with WHODAS 2.0 Self-care (r=−.289, p <.05) indicating that greater adherence is associated with lower self-care dysfunction. Medication adherence was significantly related to physical ability on the VR-12 (r=.328, p <.05), but with none of the other variables. CRT hours were not significantly related to these predictor variables.

No significant relationships were found between adherence measures and working memory (Digit Span), verbal learning and memory (HVLT), story memory (LM 1&2), executive function (Mazes, WCST), processing speed (Digit Symbol Substitution Test) or attention (IVA-2). Self-report on the BASIS-24 Cognitive Symptoms was not significantly related to adherence measures.

Predictor variables that showed some significant association from the above analyses (Age, WHODAS 2.0 Self-care and VR12 Physical Health) were entered into a stepwise linear regression analysis to predict Total Adherence. A significant model (F=4.78, df=2, p=.013) was observed with WHODAS 2.0 Self-care (adjusted Rsq=.064,) and Age (Adjusted Rsq=.072) as significant predictors (total adjusted Rsq=.136).

Predictors of self-care: Age and Self-care were the only two predictors of Total Adherence identified by the above analyses. Age was not significantly correlated with Self-care (r=−.073, p=ns) and is not a potential target for intervention. Self-care is a possible malleable clinical feature, which makes understanding its relationships to other clinical variables of interest. No relationship was found between Self-care and substance abuse or craving variables (Supplementary Table 7), but there were some significant relationships with psychiatric symptoms (Depression: r=.359, p<.01, Overall Symptoms: r=.400, p<.01) and relatedness variables (Alienation r=.400, p<.01; Insecure Attachment: r= .312, p<.05) from the BORRTI (Supplementary Tables 8 and 9). Self-care showed no significant relationships with cognitive measures (Supplementary Table 10).

Linear regression of self-care predictors: Depression, Overall Symptoms, Alienation and Insecure Attachment was entered into a stepwise linear regression and produced a significant model with Alienation as the only predictor (Adjusted Rsq=.143, F=9.32, df=1, p=.004). The other variables failed to contribute significantly to the prediction of Self-care Once Alienation was entered.

## DISCUSSION

This study showed a relatively high rate of adherence: over two-thirds of participants completed the 13-week protocol, with substantial engagement in both digital therapeutics and medication. Given the well-documented challenges of retaining individuals with AUD in treatment, these findings are promising. The remote, flexible format of the intervention likely contributed to this high engagement [25,44]. One reason may be that digital delivery reduces logistical barriers such as transportation, scheduling conflicts and stigma associated with in-person treatment. For populations often underserved by traditional healthcare systems, remote delivery methods may represent a key avenue for improving access and adherence.

This study identified several predictors of treatment adherence among patients with AUD engaged in a clinical trial involving digital cognitive training and medication. The findings highlight the importance of Self-care, as a predictor of adherence, whereas illness-specific characteristics such as AUD severity, alcohol craving, or cognitive impairment did not predict adherence. This somewhat surprising finding is in some ways good news for AUD research because it suggests that adherence may not vary by AUD characteristics. These variables are often used to stratify risk or assign patients to treatment pathways, yet in our sample, they did not differentiate between those who adhered and those who did not. This may suggest that once patients engage in a structured clinical trial, motivational and functional aspects outweigh illness severity in determining ongoing participation.

The principal finding is the relationship between better Self-care and our Total Adherence measure. This suggests that the capacity to maintain personal care routines and manage basic daily tasks may reflect an underlying motivational, organizational, or psychological readiness that also supports participation in structured interventions like cognitive remediation [26]. Participants with more ability to organize and sustain engagement in any self-care activity over time are more likely to adhere to their AUD treatment.

Medication adherence was weakly associated with younger age and better physical health status. Our finding is different from two other studies where poor adherence was associated with younger age [9,10]. It may be that the poor physical status associated with age played a role. Regarding physical health status, our finding aligns with prior literature indicating that physical comorbidities can hinder adherence [45]. Individuals with better health may have more cognitive and physical energy to engage in structured treatments, including medication regimens. However, this association was not found for CRT Hours or Total Adherence. Importantly, our data showed that despite widespread concerns about the cognitive and behavioral impairments associated with chronic alcohol use, attention, memory and executive function were not significantly related to adherence outcomes in this study.

While Self-care was the best predictor of Total Adherence, we found that psychiatric symptoms and relatedness variables were predictors of Self-care. Regression analysis suggests that Alienation captures the most variance in Self-care. This leads to a potential model in which participants who feel profound Alienation, including distrust and a belief that relationships cannot be gratifying, may not develop the therapeutic alliance with medical professionals that is so essential for sustaining self-care.

Our results suggest that future strategies to boost adherence should focus on helping patients establish daily structure, offering reminders and integrating self-care practices into treatment routines. Making special efforts to build and sustain positive therapeutic relationships may be especially relevant. Examples of self-care practices that may promote adherence include mindfulness-based self-care, self-monitoring of alcohol use, self-management and nonjudgmental approaches to self and others [26].

## CONCLUSION

In conclusion, our exploratory study suggests that treatment adherence in AUD treatment trials involving digital therapeutics and medication may not be directly influenced by illness characteristics, but primarily by the individuals self-reported capacity for self-care. Self-reported Alienation was the best predictor of self-care, suggesting that individuals who distrust relationships and may be more isolated may have greater difficulty following medical self-care. These findings suggest that supporting patients in their basic daily functioning and fostering a therapeutic alliance may be a critical step toward enhancing treatment engagement and improving long-term outcomes. As healthcare increasingly embraces digital and remote delivery, it will be important to understand factors that influence treatment adherence so that providers can identify those less likely to follow-through on treatment and to develop person-centered strategies to enhance therapeutic alliance even while providing digital care.

These findings enhance our understanding of treatment adherence in AUD clinical trials. By identifying factors unrelated to illness severity, but tied to general self-care ability, we can design better, more accessible interventions to support patients in their recovery journey.

## LIMITATIONS

There are several limitations to this study. The small sample size reduced statistical power so that only moderate to large effects could be detected and we did not correct for multiple comparisons. The blind, which prevents access to treatment arm information, restricted our statistical comparisons, so we do not know if sham CRT or placebo had the same adherence as the active conditions. Medication adherence was based on self-report with corroboration from Pharmacy about picking up their prescribed medication. This method may affect validity but was chosen because the weekly interviews were performed by phone, thus making pill counts or watching the participant take the medications more difficult. On the other hand, we have an exact count of the time spent in CRT from Posit Science, even if we cannot know the effort of the participant in performing the tasks. Although we believe our baseline measures are representative and comprehensive, it is possible that we missed some individual characteristic that might have been predictive. Finally, findings may not generalize to other populations and other digital therapeutics for different conditions.

## Supplementary Material

Supplementary file
