# Supplementary material for: Predictors of Treatment Adherence in a Randomized Clinical Trial of Digital Therapeutics with Pharmacology for Alcohol Use Disorder (AUD)
Source: J Clin Trials. Author manuscript; Available in PMC 2025 Dec 27. (PMC12742581)
Supplement: Supplementary file [file NIHMS2108122-supplement-Supplementary_file.pdf]

Journal of Clinical Trials
ISSN: 2167-0870
OPEN ACCESS Freely available online

Research Article

Predictors of Treatment Adherence in A Randomized Clinical Trial of Digital Therapeutics with Pharmacology for Alcohol Use Disorder (AUD)

Morris D. Bell[1,2*], Yarani Gonzalez[1,2], Brian Pittman[1,2], Gihyun Yoon[1,2]

[1]*Department of Psychiatry, Yale School of Medicine, New Haven, CT, USA;* [2]*VA Connecticut Healthcare System, West Haven, CT, USA*

Supplementary Table 1: Descriptive statistics.

Variable	Mean	Standard Deviation	N
Education (years)	14.69	3.13	52
Age	46.38	14.9	52
Total Adherence	3.29	2.25	49
CRT Hours	29.49	27.9	49
Raw Pill Count	48.78	38.81	49
WTAR Standard score	109.88	12.78	51
Alcohol Use (months)	244.37	180.53	52
Cravings (raw Penn scale)	17.92	6.77	49
Alcohol Use (BASIS-24)	1.72	0.76	51
BASIS-24 Depression/Functioning	1.23	0.76	51
BASIS-24 Interpersonal Problems	1.35	1.01	51
BASIS-24 Psychotic Symptoms	0.24	0.41	51
BASIS-24 Emotional Lability	1.34	0.89	51
BASIS-24 Self-harm	0.1	0.37	51
Overall BASIS-24 Score	1.12	0.57	51
BORRTI Alienation t-score	52.04	11.19	51
BORRTI Insecure attachment t-score	47.61	7.41	51
BORRTI Egocentricity t-score	52.98	9.6	51
BORRTI Social incompetence t-score	47.25	10.16	51
BORRTI Reality distortion t-score	49.61	7.26	51
BORRTI Uncertainty of perception t-score	46.25	9.17	51
BORRTI Hallucinations and delusions t-score	46.16	7.56	51
WHODAS Cognition %	20.14	16.26	51
WHODAS Mobility %	15.92	19.06	51
WHODAS Self-care %	8.04	12.81	51

Correspondence to: Morris D. Bell, Department of Psychiatry, Yale School of Medicine, New Haven, CT, USA, E-mail: morris.bell@yale.edu

Received: 07-Jul-2025, Manuscript No. JCTR-25-37966; **Editor assigned:** 09-Ju1-2025, PreQC No. JCTR-25-37966 (PQ); **Reviewed:** 23-Jul-2025, QC No. JCTR-25-37966; **Revised:** 30-Jul-2025, Manuscript No. JCTR-25-37966 (R); **Published:** 08-Aug-2025, DOI: 10.35248/2167-0870.25.15.597

Citation: Bell MD, Gonzalez Y, Pittman B, Yoon G (2025). Predictors of Treatment Adherence in A Randomized Clinical Trial of Digital Therapeutics with Pharmacology for Alcohol Use Disorder (AUD). J Clin Trials. 15:597.

Copyright: © 2025 Bell MD, et al. This is an open-access article distributed under the terms of the Creative Commons Attribution License, which permits unrestricted use, distribution and reproduction in any medium, provided the original author and source are credited.

WHODAS Getting Along %	23.1	19.76	51
WHODAS Household %	28.63	25.54	51
WHODAS Participation %	22.51	15.99	51
WHODAS Overall Total %	20.06	14.21	51
VR-12 Physical t-score	46.08	14.94	51
VR-12 Emotional t-score	45.8	17.87	51
HVLT Total Recall t-score	41.63	10.27	51
HVLT Delayed Recall t-score	43.7	11.25	50
Digit Symbols t-score	46.63	8.28	51
Digit Span t-score	47.39	12.14	51
Mazes t-score	53.76	9.32	51
Logical Memory 1 t-score	51.8	11.26	51
Logical Memory 2 t-score	54.35	10.58	49
IVA Full Scale q-score	89.13	26.38	46
WCST PR error t-score	45.45	8.15	47
WCST Conceptual response t-score	43.6	9.79	48
WCST Categories Raw Score	3	1.62	48

Note: CRT: Cognitive Remediation Therapy; WTAR: Wechsler Test of Adult Reading; BASIS-24: Behavior And Symptom Identification Scale; BORRTI: Bell Object Relations and Reality Testing Inventory; WHODAS: WHO Disability Assessment Schedule; VR-12:Veterans Rand 12-Item Health Survey; HVLT: Hopkins Verbal Learning Test Revised; IVA: Integrated Visual and Auditory; CPT: Continuous Performance Test; WCST: Wisconsin Card Sorting Test

Supplementary Table 1a: Descriptive statistics-Demographic information and alcohol use.

Variable	Mean	Standard Deviation	N
Education (years)	14.69	3.128	52
Age	46.38	14.9	52
Total Adherence	3.29	2.245	49
CRT Hours	29.49	27.89	49
Raw Pill Count	48.78	38.8	49
WTAR Standard Score	109.88	12.79	51
Alcohol Use (months)	244.37	180.53	52
Cravings (raw Penn scale)	17.92	6.77	49
Alcohol Use (BASIS 24)	1.72	0.76	51

Note: CRT: Cognitive Remediation Therapy; WTAR: Wechsler Test of Adult Reading; BASIS-24: Behavior And Symptom Identification Scale

Supplementary Table 1b: Descriptive Statistics-Behavior And Symptom Identification Scale (BASIS 24) (N=51).

Variable	Mean	Standard Deviation	N
Alcohol and Drug Use	1.72	0.76	51
Depression/Functioning	1.23	0.76	51
Interpersonal Problems	1.35	1.01	51
Psychotic Symptoms	0.24	0.41	51
Emotional Lability	1.34	0.89	51
Self-harm	0.1	0.37	51
Overall BASIS-24 Score	1.12	0.57	51

Note: BASIS-24: Behavior And Symptom Identification Scale

Supplementary Table 1c: Descriptive Statistics-Bell Object Relations Reality Testing Inventory (BORRTI) (N=51).

Variable	Mean	Standard Deviation	N
BORRTI Alienation t-score	52.04	11.19	51
BORRTI Insecure Attachment t-score	47.61	7.41	51
BORRTI Egocentricity t-score	52.98	9.6	51
BORRTI Social Incompetence t-score	47.25	10.16	51
BORRTI Reality Distortion t-score	49.61	7.26	51
BORRTI Uncertainty of Perception t-score	46.25	9.17	51
BORRTI Hallucinations and Delusions t-score	46.16	7.56	51

Note: BORRTI: Bell Object Relations and Reality Testing Inventory

Supplementary Table 1d: Descriptive Statistics-WHO Disability Assessment Schedule (WHODAS 2.0) and Veterans Rand 12-Item Health Survey (VR-12) (N=51).

Variable	Mean	Standard Deviation	N
WHODAS Cognition %	20.14	16.26	51
WHODAS Mobility %	15.92	19.06	51
WHODAS Self-care %	8.04	12.81	51
WHODAS Getting Along %	23.1	19.76	51
WHODAS Household %	28.63	25.54	51
WHODAS Participation %	22.51	15.99	51
WHODAS Overall Total %	20.06	14.21	51
VR-12 Physical t-score	46.08	14.94	51
VR-12 Emotional t-score	45.8	17.87	51

Note: WHODAS WHO Disability Assessment Schedule; VR-12:Veterans Rand 12-Item Health Survey

Supplementary Table 1e: Descriptive Statistics-Cognitive testing.

Variable	Mean	Standard Deviation	N
HVLT Total Recall t-score	41.63	10.27	51
HVLT Delayed Recall t-score	43.7	11.25	50
Digit Symbols t-score	46.63	8.28	51
Digit Span t-score	47.39	12.14	51
Mazes t-score	53.76	9.32	51
Logical Memory 1 t-score	51.8	11.26	51
Logical Memory 2 t-score	54.35	10.58	49
IVA Full Scale q-score	89.13	26.38	46
WCST PR error t-score	45.45	8.15	47
WCST Conceptual Response t-score	43.6	9.79	48
WCST Categories Raw Score	3	1.624	48

Note: HVLT: Hopkins Verbal Learning Test Revised; IVA: Integrated Visual and Auditory (IVA) Continuous Performance Test; WCST: Wisconsin Card Sorting Test

Supplementary Table 2: Alcohol Use and Craving Correlations with Adherence Measures.

Variable		Total Adherence	Raw Pill Counts	CRT Hours
Total Intoxication Alcohol Use in Months	Correlation	-0.207	-.338*	-0.123
	Sig. (2-tailed)	0.153	0.018	0.4
	N	49	49	49
Penn Craving Raw Score	Correlation	0.166	0.231	0.05
	Sig. (2-tailed)	0.254	0.114	0.736
	N	48	48	48
BASIS-24 Alcohol and Drug Use	Correlation	0.085	0.069	0.074
	Sig. (2-tailed)	0.562	0.635	0.611
	N	49	49	49

Note: **Correlation is significant at the 0.01 level (2-tailed); *Correlation is significant at the 0.05 level (2-tailed); BASIS-24: Behavior And Symptom Identification Scale

Supplementary Table 3: Symptoms Correlations with Adherence Measures (N=49).

Variable		Total Adherence	Raw Pill Counts	CRT Hours
Total Adherence	Correlation	1	0.848**	.872**
	Sig. (2-tailed)		<.001	<.001
Raw Pill Counts	Correlation	0.848**	1	.556**
	Sig. (2-tailed)	<.001		<.001
CRT Hours	Correlation	.872**	.561**	1
	Sig. (2-tailed)	<.001	<.001	
BASIS-24 Depression/ Functioning	Correlation	-0.005	-0.041	-0.129
	Sig. (2-tailed)	0.975	0.782	0.377
BASIS-24 Interpersonal Problems	Correlation	-0.084	-0.031	-0.156
	Sig. (2-tailed)	0.564	0.831	0.285
BASIS-24 Psychotic Symptoms	Correlation	-0.016	-0.178	0.037
	Sig. (2-tailed)	0.914	0.22	0.801
BASIS-24 Emotional Lability	Correlation	-0.061	-0.118	-0.038
	Sig. (2-tailed)	0.675	0.419	0.794
BASIS-24 Self-harm	Correlation	0.109	0.18	0.028
	Sig. (2-tailed)	0.458	0.216	0.849
BASIS-24 Overall	Correlation	-0.039	-0.066	-0.123
	Sig. (2-tailed)	0.784	0.652	0.398

Note: **Correlation is significant at the 0.01 level (2-tailed); *Correlation is significant at the 0.05 level (2-tailed); CRT: Cognitive Remediation Therapy; BASIS-24: Behavior And Symptom Identification Scale

Supplementary Table 4: Bell Object Relations Reality Testing Inventory (BORRTI) Correlations with Adherence Measures (N=49).

Variable		Total Adherence	Raw Pill Counts	CRT Hours
BORRTI Alienation t-score	Correlation	-0.049	-0.077	-0.173
	Sig. (2-tailed)	0.737	0.598	0.235
BORRTI Insecure Attachment t-score	Correlation	0.125	0.117	0.022
	Sig. (2-tailed)	0.394	0.422	0.882
BORRTI Egocentricity t-score	Correlation	0.008	0.032	-0.069
	Sig. (2-tailed)	0.956	0.828	0.636

BORRTI Social Incompetence t-score	Correlation	0.108	0.128	0.026
	Sig. (2-tailed)	0.461	0.379	0.857
BORRTI Reality Distortion t-score	Correlation	0.022	0.026	-0.06
	Sig. (2-tailed)	0.882	0.86	0.681
BORRTI Uncertainty of Perception t-score	Correlation	-0.134	-0.059	-0.162
	Sig. (2-tailed)	0.357	0.688	0.266
BORRTI Hallucinations and Delusions t-score	Correlation	0.021	-0.068	0.066
	Sig. (2-tailed)	0.888	0.641	0.653

Note: **Correlation is significant at the 0.01 level (2-tailed); *Correlation is significant at the 0.05 level (2-tailed); BORRTI: Bell Object Relations and Reality Testing Inventory

Supplementary Table 5: WHO Disability Assessment Schedule (WHODAS 2.0) and Veterans Rand 12-Item Health Survey (VR-12) Correlations with Adherence Measures (N=49).

Variable		Total Adherence	Raw Pill Counts	CRT Hours
WHODAS Cognition %	Correlation	-0.172	-0.139	-0.192
	Sig. (2-tailed)	0.236	0.339	0.186
WHODAS Mobility %	Correlation	-0.218	-0.246	-0.272
	Sig. (2-tailed)	0.133	0.088	0.059
WHODAS Self-care %	Correlation	-.289*	-0.192	-0.271
	Sig. (2-tailed)	0.044	0.186	0.059
WHODAS Getting Along %	Correlation	-0.121	-0.129	-0.159
	Sig. (2-tailed)	0.407	0.377	0.275
WHODAS Household %	Correlation	-0.26	-0.178	-0.221
	Sig. (2-tailed)	0.072	0.22	0.128
WHODAS Participation %	Correlation	-0.141	-0.113	-0.145
	Sig. (2-tailed)	0.333	0.439	0.32
WHODAS Overall Total %	Correlation	-0.247	-0.172	-0.234
	Sig. (2-tailed)	0.087	0.236	0.105
VR-12 Physical t-score	Correlation	0.256	.328*	0.192
	Sig. (2-tailed)	0.076	0.021	0.187
VR-12 Emotional t-score	Correlation	0.051	-0.047	0.059
	Sig. (2-tailed)	0.729	0.747	0.685

Note: **Correlation is significant at the 0.01 level (2-tailed); *Correlation is significant at the 0.05 level (2-tailed); WHODAS WHO Disability Assessment Schedule; VR-12:Veterans Rand 12-Item Health Survey

Supplementary Table 6: Cognitive Testing Correlations with Adherence Measures (N=49).

Variable		Total Adherence	Raw Pill Counts	CRT Hours
WHODAS Cognition %	Correlation	-0.172	-0.139	-0.192
	Sig. (2-tailed)	0.236	0.339	0.186
HVLT Total Recall t-score	Correlation	0.086	0.084	0.087
	Sig. (2-tailed)	0.559	0.564	0.551
HVLT Delayed Recall t-score	Correlation	0.093	0.126	0.077
	Sig. (2-tailed)	0.531	0.392	0.604
Digit Symbol t-core	Correlation	0.103	0.156	0.044
	Sig. (2-tailed)	0.483	0.284	0.763

Digit Span t-score	Correlation	0.175	0.187	0.187
	Sig. (2-tailed)	0.229	0.198	0.198
Mazes t-score	Correlation	0.074	0.061	0.121
	Sig. (2-tailed)	0.615	0.676	0.406
Logical Memory 1 t-score	Correlation	0.093	0.088	0.035
	Sig. (2-tailed)	0.527	0.548	0.813
Logical Memory 2 t-score	Correlation	0.003	0.035	0.024
	Sig. (2-tailed)	0.986	0.815	0.871
IVA Full Scale q-score	Correlation	0.09	0.012	-0.027
	Sig. (2-tailed)	0.557	0.937	0.859
WCST PR err t-score	Correlation	0.095	0.039	0.065
	Sig. (2-tailed)	0.529	0.796	0.67
WCST Conceptual Response t-score	Correlation	0.235	0.229	0.14
	Sig. (2-tailed)	0.111	0.122	0.35
WCST Categories Raw Score	Correlation	0.223	0.213	-0.175
	Sig. (2-tailed)	0.132	0.151	0.24

Note: **Correlation is significant at the 0.01 level (2-tailed);*Correlation is significant at the 0.05 level (2-tailed); WHODAS WHO Disability Assessment Schedule; HVLT: Hopkins Verbal Learning Test Revised; IVA: Integrated Visual and Auditory (IVA) Continuous Performance Test; WCST: Wisconsin Card Sorting Test

Supplementary Table 7: Self-Care Correlations.

Variable		Age	WHODAS Self-care %	Total Intoxication (Alcohol Use in Months)	Penn Craving Raw Score	BASIS 24 Alcohol and Drug Use
Age	Correlation	1	-0.059	.520**	-0.008	-0.017
	Sig. (2-tailed)		0.683	<.001	0.954	0.908
	N	52	51	52	49	51
WHODAS Self-Care%	Correlation	-0.059	1	-0.091	0.074	-0.031
	Sig. (2-tailed)	0.683		0.526	0.612	0.827
	N	51	51	51	49	51
Total Intoxication Alcohol Use in Months	Correlation	.520**	-0.091	1	0.128	-0.049
	Sig. (2-tailed)	<.001	0.526		0.379	0.732
	N	52	51	52	49	51
Penn Craving Raw Score	Correlation	-0.008	0.074	0.128	1	.287*
	Sig. (2-tailed)	0.954	0.612	0.379		0.046
	N	49	49	49	49	49
BASIS 24 Alcohol and Drug Use	Correlation	-0.017	-0.031	-0.049	.287*	1
	Sig. (2-tailed)	0.908	0.827	0.732	0.046	
	N	51	51	51	49	51

Note: **Correlation is significant at the 0.01 level (2-tailed); *Correlation is significant at the 0.05 level (2-tailed); BASIS-24: Behavior And Symptom Identification Scale; WHODAS WHO Disability Assessment Schedule

Supplementary Table 8: Self-care and Behavior And Symptom Identification Scale (BASIS 24) Symptom Correlates (N=51).

Variable		WHODAS Self-Care %	Depression/ Functioning	Interpersonal Problems	Psychotic Symptoms	Emotional Lability	Self-Harm	Overall
WHODAS Self-Care %	Correlation	1	.359**	0.259	0.123	.307*	0.111	.400**
	Sig. (2-tailed)		0.01	0.067	0.389	0.029	0.438	0.004
Depression/ Functioning	Correlation	.359**	1	0.243	.400**	.633**	.461**	.927**
	Sig. (2-tailed)	0.01		0.085	0.004	<.001	<.001	<.001
Interpersonal Problems	Correlation	0.259	0.243	1	.472**	0.247	0.148	.511**
	Sig. (2-tailed)	0.067	0.085		<.001	0.08	0.298	<.001
Psychotic Symptoms	Correlation	0.123	.400**	.472**	1	.593**	0.234	.596**
	Sig. (2-tailed)	0.389	0.004	<.001		<.001	0.098	<.001
Emotional Lability	Correlation	.307*	.633**	0.247	.593**	1	0.153	.767**
	Sig. (2-tailed)	0.029	<.001	0.08	<.001		0.284	<.001
Self-Harm	Correlation	0.111	.461**	0.148	0.234	0.153	1	.460**
	Sig. (2-tailed)	0.438	<.001	0.298	0.098	0.284		<.001
Overall	Correlation	.400**	.927**	.511**	.596**	.767**	.460**	1
	Sig. (2-tailed)	0.004	<.001	<.001	<.001	<.001	<.001	

Note: **Correlation is significant at the 0.01 level (2-tailed); *Correlation is significant at the 0.05 level (2-tailed); BASIS-24: Behavior And Symptom Identification Scale; WHODAS WHO Disability Assessment Schedule

Supplementary Table 9: Self-care and Bell Object Relations and Reality Testing Inventory (BORRTI) Symptom Correlates (N=51).

Variable		WHODAS Self-care %	Alienation t-score	Insecure Attachment t-score	Ego-centricity t-score	Social Incompetence t-score	Reality Distortion t-score	Uncertainty of Perception t-Score	Hallucinations and Delusions t-score
WHODAS Self-Care %	Correlation	1	.400**	.312*	-0.015	0.087	0.196	0.219	0.094
	Sig. (2-tailed)		0.004	0.026	0.917	0.544	0.168	0.123	0.511
Alienation t-score	Correlation	.400**	1	.542**	0.248	.363**	.486**	.623**	0.259
	Sig. (2-tailed)	0.004		<.001	0.079	0.009	<.001	<.001	0.066
Insecure Attachment t-score	Correlation	.312*	.542**	1	.351*	.494**	.314*	.472**	0.204
	Sig. (2-tailed)	0.026	<.001		0.011	<.001	0.025	<.001	0.151
Egocentricity t-score	Correlation	-0.015	0.248	0.351	1	-0.046	.472**	.282*	0.195
	Sig. (2-tailed)	0.917	0.079	0.011		0.749	<.001	0.045	0.171
Social Incompetence t-score	Correlation	0.087	.363**	.494**	-0.046	1	0.163	0.228	0.205
	Sig. (2-tailed)	0.544	0.009	<.001	0.749		0.252	0.107	0.15
Reality Distortion t-score	Correlation	0.196	.486**	.314*	.472*	0.163	1	.432**	0.123
	Sig. (2-tailed)	0.168	<.001	0.025	<.001	0.252		0.002	0.391
Uncertainty of Perception t-score	Correlation	0.219	.623**	.472**	.282*	0.228	.432**	1	.360*
	Sig. (2-tailed)	0.123	<.001	<.001	0.045	0.107	0.002		0.01
Hallucinations and Delusions t-score	Correlation	0.094	0.259	0.204	0.195	0.205	0.123	.360**	1
	Sig. (2-tailed)	0.511	0.066	0.151	0.171	0.15	0.391	0.01	

Note: **Correlation is significant at the 0.01 level (2-tailed); *Correlation is significant at the 0.05 level (2-tailed); BORRTI: Bell Object Relations and Reality Testing Inventory; WHODAS WHO Disability Assessment Schedule

Supplementary Table 10: Self-care and Cognitive Variable Correlates.

Variable		WHODAS Self-care %	HVLT Total Recall t-score	HVLT Delayed Recall t-score	Digit Symbols t-score	Digit Span t-score	Mazes t-score	Logical Memory 1 t-score	Logical Memory 2 t-score	IVA Full Scale q	WCST PR Error t-score	WCST Conceptual t-score	WCST Categories score
WHODAS Self-Care %	Correlation	1	0.032	0.103	-0.124	-0.026	-0.031	0.015	-0.02	0.033	0.016	-0.116	-0.171
	Sig. (2-tailed)		0.822	0.476	0.386	0.857	0.83	0.915	0.889	0.828	0.916	0.431	0.246
	N	51	51	50	51	51	51	51	49	46	47	48	48
HVLT Total Recall t-score	Correlation	0.032	1	.779**	0.236	.489**	0.173	.499**	.385**	.421**	0.14	.372**	.328*
	Sig. (2-tailed)	0.822		<.001	0.095	<.001	0.224	<.001	0.006	0.004	0.349	0.009	0.023
	N	51	51	50	51	51	51	51	49	46	47	48	48
HVLT Delayed Recall t-score	Correlation	0.103	.779**	1	0.243	.371**	0.264	.465**	.446**	0.269	0.127	.321*	0.257
	Sig. (2-tailed)	0.476	<.001		0.09	0.008	0.064	<.001	0.002	0.074	0.399	0.028	0.081
	N	50	50	50	50	50	50	50	48	45	46	47	47
Digit Symbols t-score	Correlation	-0.124	0.236	0.243	1	0.197	.381**	.319*	0.177	.485**	-0.056	0.169	.344*
	Sig. (2-tailed)	0.386	0.095	0.09		0.166	0.006	0.023	0.223	<.001	0.708	0.25	0.017
	N	51	51	50	51	51	51	51	49	46	47	48	48
Digit Span t-score	Correlation	-0.026	.489**	.371**	0.197	1	0.117	.627**	.452**	.313*	0.239	.353*	.317*
	Sig. (2-tailed)	0.857	<.001	0.008	0.166		0.412	<.001	0.001	0.034	0.106	0.014	0.028
	N	51	51	50	51	51	51	51	49	46	47	48	48
Mazes t-score	Correlation	-0.031	0.173	0.264	.381**	0.117	1	.391**	.420**	.334*	0.022	0.143	0.264
	Sig. (2-tailed)	0.83	0.224	0.064	0.006	0.412		0.005	0.003	0.023	0.882	0.334	0.07
	N	51	51	50	51	51	51	51	49	46	47	48	48

Logical Memory 1 t-score	Correlation	0.015	.499**	.465**	.319*	.627**	.391**	1	.814**	0.332	0.073	.356*	.459**
	Sig. (2-tailed)	0.915	<.001	<.001	0.023	<.001	0.005		<.001	0.024	0.628	0.013	0.001
	N	51	51	50	51	51	51	51	49	46	47	48	48
Logical Memory 2 t-score	Correlation	-0.02	.385**	.446**	0.177	.452**	.420**	.814**	1	0.145	0.131	.310*	.327*
	Sig. (2-tailed)	0.889	0.006	0.002	0.223	0.001	0.003	<.001		0.347	0.391	0.036	0.026
	N	49	49	48	49	49	49	49	49	44	45	46	46
IVA Full Scale q-score	Correlation	0.033	.421**	0.269	.485**	.313*	.334*	.332*	0.145	1	0.12	0.157	0.262
	Sig. (2-tailed)	0.828	0.004	0.074	<.001	0.034	0.023	0.024	0.347		0.431	0.303	0.083
	N	46	46	45	46	46	46	46	44	46	45	45	45
WCST PR error t-score	Correlation	0.016	0.14	0.127	-0.056	0.239	0.022	0.073	0.131	0.12	1	.720**	.526**
	Sig. (2-tailed)	0.916	0.349	0.399	0.708	0.106	0.882	0.628	0.391	0.431		<.001	<.001
	N	47	47	46	47	47	47	47	45	45	47	47	47
WCST Conceptual Response t-score	Correlation	-0.116	.372**	.321*	0.169	.353*	0.143	.356*	.310*	0.157	.720**	1	.896**
	Sig. (2-tailed)	0.431	0.009	0.028	0.25	0.014	0.334	0.013	0.036	0.303	<.001		<.001
	N	48	48	47	48	48	48	48	46	45	47	48	48
WCST Categories Raw Score	Correlation	-0.171	.328*	0.257	.344*	.317*	0.264	.459**	.327*	0.262	.526**	.896**	1
	Sig. (2-tailed)	0.246	0.023	0.081	0.017	0.028	0.07	0.001	0.026	0.083	<.001	<.001	
	N	48	48	47	48	48	48	48	46	45	47	48	48

Note: **Correlation is significant at the 0.01 level (2-tailed); *Correlation is significant at the 0.05 level (2-tailed); WHODAS WHO Disability Assessment Schedule; HVLT: Hopkins Verbal Learning Test Revised; IVA: Integrated Visual and Auditory (IVA) Continuous Performance Test; WCST: Wisconsin Card Sorting Test
